# Comparing healthcare quality: A common framework for both ordinal and cardinal data with an application to primary care variation in England

**DOI:** 10.1002/hec.4597

**Published:** 2022-08-28

**Authors:** Paul Allanson, Richard Cookson

**Affiliations:** ^1^ UDSB University of Dundee Dundee UK; ^2^ Centre for Health Economics University of York York UK

**Keywords:** comparative quality evaluation, England, healthcare variation, ordinal data, primary care services

## Abstract

The paper proposes a framework for comparing the quality of healthcare providers and assessing the variation in quality between them, which is directly applicable to both ordinal and cardinal quality data on a comparable basis. The resultant measures are sensitive to the full distribution of quality scores for each provider, not just the mean or the proportion meeting some binary quality threshold, thereby making full use of the multicategory response data increasingly available from patient experience surveys. The measures can also be standardized for factors such as age, sex, ethnicity, health and deprivation using a distribution regression model. We illustrate by measuring the quality of primary care services in England in 2019 using three different sources of publicly available, general practice‐level information: multicategory response patient experience data, ordinal inspection ratings and cardinal clinical achievement scores. We find considerable variation at both local and regional levels using all three data sources. However, the correlation between the comparative quality indices calculated using the alternative data sources is weak, suggesting that they capture different aspects of general practice quality.

## 1 INTRODUCTION

Comparing the quality of healthcare providers and measuring the degree of variation in quality are major policy concerns in many countries (Busse et al., 2019), with patients in England commonly said to face a “postcode lottery” in which their choice of healthcare provider and hence the quality of care they can expect to receive is determined by where they live. Making quality comparisons between healthcare providers or geographical areas is a routine exercise based on quantitative indicators of structure, process and outcome quality (Mainz, 2003), with the degree of variation captured using summary statistics such as the extremal quotient, coefficient of variation and systematic component of variation (Ibáñez et al., 2009). However, these summary statistics are only appropriate for quality indicators measured on a cardinal scale, such as staff to patient ratios, proportions of patients receiving indicated treatment and risk‐adjusted mortality rates. Nowadays, cardinal quality indicators are increasingly being supplemented by multicategory response information from patient experience surveys in which, importantly, respondents are typically asked to assess their quality of care by choosing between one of several ranked categories (e.g., very poor, poor, OK, good, very good). For example, England initiated a national patient survey program in 2001 (DeCourcy et al., 2012), with surveys now regularly conducted of patient experience in a range of primary and secondary care settings (NHS England, 2021).

A critical limitation of this patient‐reported data for the summary evaluation of both the performance of individual healthcare providers and the variation between them is its qualitative or ordinal nature. In particular, the mean is not well defined for polytomous categorical response data, which in turn severely restricts the choice of dispersion measures. A common workaround has been to impose some numerical, perhaps latent, scale on the ordinal data, but this chosen scale is essentially arbitrary and different scales can yield substantially different results. For example, the resultant ranking of healthcare providers by mean quality levels will not in general be robust to simple monotonic transformations of the chosen scale (cf. Bond & Lang, 2019) and this non‐robustness problem extends to measures of variation that are a function of the mean (Allison & Forster, 2004). Another popular option is to collapse the number of categories to yield a binary 0/1 indicator that is amenable to analysis in terms of the proportion of patients reporting good (as opposed to not good) care (see e.g., Bruyneel et al., 2017). However, the choice of cutoff is again arbitrary yet impactful, and information is also inevitably discarded in the process. Neither of these standard approaches is therefore entirely satisfactory despite their widespread use in practice.

The main contributions of this paper are twofold. First, we propose a quality assessment framework that is directly applicable to ordinal as well as cardinal quality indicators, without the need to first convert ordinal indicators into a cardinal scale such as the proportion meeting a binary threshold. For this purpose, we build directly on the methods used by Allanson (2021) to assess regional variation in ordinal indicators of health on the basis of the statistical preference criterion (De Schuymer et al., 2003; Montes et al., 2015), showing how this approach can be applied to cardinal as well as ordinal indicators and providing a novel application to the context of healthcare performance evaluation motivated by the notion that patients face a postcode lottery in healthcare provision. Specifically, we make use of information about the care quality profiles or distributions of all healthcare providers serving some population of interest to provide intelligible measures of both the comparative quality of each provider and the variation in quality between them. The comparative quality of a provider is defined as the difference in the chances that the quality of care received by a randomly chosen patient treated by that provider will be better rather than worse than that received by a randomly chosen patient from the population as a whole. The measure of variation is equal to the average absolute difference in the chances that the quality of care received by patients will be better rather than worse as a result of being treated by one provider rather than another, leading us to call it the “lottery” index. This index will take a minimum value of zero if all quality profiles are identical such that there is no difference in the chances that a randomly chosen patient treated by one provider will receive better rather than worse care than one treated by another. Conversely, it will take a maximum value of one if the quality of care provided by any one provider is certain to be either strictly better or strictly worse than that provided by any other, which will only be the case for non‐overlapping quality profiles. The intuition and mathematics behind our measures are set out in detail in the assessment framework section below.

Second, we show how our assessment framework can generate useful new insights into the performance of healthcare systems by applying it to three different practice‐level indicators of the quality of primary care services in England—categorical response data from the annual GP Patient Survey (GPPS), ordinal inspection ratings from the Care Quality Commission (CQC), and cardinal measures of process quality from the Quality and Outcomes Framework (QOF)—all of which are published in searchable online databases to help inform patients' choices. Primary care services in England are delivered through general practices (“practices” hereafter) with the average practice responsible for the care of about 7000 adult patients. All practices are a member of one of nearly 200 Clinical Commissioning Groups (CCGs), which are responsible for the planning and commissioning of health care services for their local populations. We therefore examine variation in quality both between practices and between CCGs, where our analysis is most likely to be of interest to healthcare managers and policymakers responsible for the delivery of services at the population level rather than to individual patients looking to choose a practice that will meet their own personal care needs and preferences.

The CQC, the independent regulator of health and social care service providers, reported wide variation between practices in the mean number of full‐time equivalent general practitioners (GPs) per head of registered population in 2018/19, with the geographical concentration of poor quality care, as shown by inspection ratings, making it difficult for people living in some areas to access good care (CQC 2019b, pp. 19, 20). NHS England and Ipsos MORI (2019, p. 10) report considerable variation across individual CCGs in the proportion of patients describing their practice as either fairly or very good in the 2019 GPPS, ranging from 69.1% to 92.1%. Patients were given the right to choose their practice in 2015, with the aim of improving the quality of access to GP services, although practices are not bound to accept patients living outside their catchment area. Santos et al. (2017) investigate patients' choice of family doctor and show that individuals are more likely to choose practices with higher standards of care as measured by their total QOF score across all achievement indicators, trading off practice quality against distance.

Policy concern about variation in the quality of healthcare services relates specifically to that part of the variation not warranted by differences in patient need or preferences. Accordingly, measures of healthcare performance are often standardized with the aim of identifying this unwarranted variation by controlling for the effects of differences in patient characteristics not under the control of providers such as age, sex, ethnicity, health and deprivation (see e.g., Dartmouth Atlas of Healthcare; Public Health England, 2015). To investigate the impact of standardization on variation in primary care quality at the CCG level we report results based on both raw and indirectly standardized practice quality profiles, where the latter are what would be expected if quality outcomes conditional upon socio‐demographic characteristics were the same in each practice as in England as a whole.

The main empirical analysis is based on data from the 2019 English GPPS questionnaire, which was sent out to more than 2 million people asking for feedback on their experiences. Practice‐level experience data, weighted by age and gender to resemble the population of eligible patients within each practice, are reported for nearly 7000 practices across 195 CCGs. We make use of the data on the proportions of patients in each practice reporting their overall experience as very poor, fairly poor, neither good nor poor, fairly good, and very good to explore the variation in primary care quality both between practices within each CCG and between CCGs in England. We also investigate the variation in primary care quality between CCGs using the CQC overall rating and total QOF score for each practice to see if these indicators provide ordinally equivalent information to the GPPS on some common latent “primary care quality” characteristic. Analysis of the CQC and QOF data is restricted to the CCG level because the practice‐level quality profiles for these indicators consist simply of a single overall rating or score. Comparative quality indices are calculated for all practices and CCGs using the GPPS data, and for all CCGs using the CQC and QOF data.

The remainder of the paper is organized as follows. The next section introduces the conceptual framework, motivating the definitions of the comparative quality and lottery indices and outlining the indirect standardization procedure. Section 3 discusses the various sources of data on practice quality which are employed in the empirical study, with the results presented in Section 4. The final section provides a discussion of the findings and concludes.

## 2 ASSESSMENT FRAMEWORK

The basic building block of our assessment framework is the comparative evaluation of the quality profiles of pairs of healthcare providers (i.e., practices or CCGs) based on information about the care quality profile or distribution of each healthcare provider. We start with a simple numerical example to provide the intuition behind the approach, before turning to the general mathematical formulation and properties of the comparative quality and lottery indices. Finally, we outline the indirect standardization procedure.

### 2.1 Assessing pairwise quality differences

**FIGURE 1:**
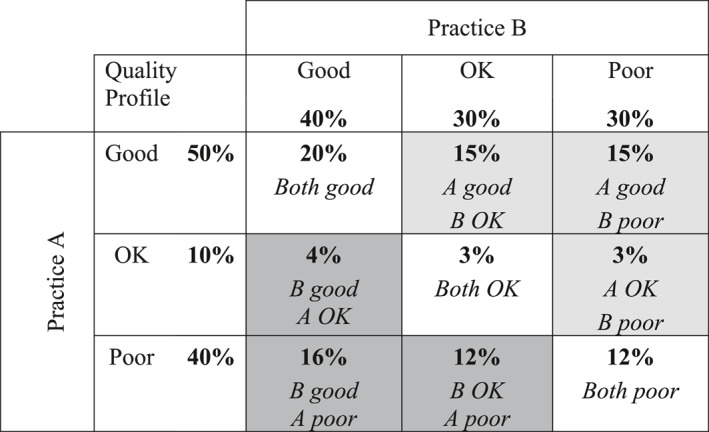
Two practice lottery example. The light and dark gray shaded boxes show respectively the proportion of draws in which care is better in A than B and vice versa, assuming care quality for each practice is chosen independently and at random from the quality profile for that practice

Figure 1 provides an example in which the quality profiles for two practices, A and B, are given as the proportion of patients in each practice who report their care as either “poor”, “OK”, or “good”—a three‐valued ordinal scale. We first note that neither conversion to a numerical scale nor dichotomization of the categories leads to a robust ranking of the quality profiles of the two practices. With numerical scaling, the mean quality of the two practices will be the same if the response options are assumed to be evenly spaced, being equal to 2.1 if the categories are scored 1, 2 and 3. But A has the higher mean if the distance between good and OK is greater than between OK and poor, whereas B has the higher mean if the opposite is the case. With dichotomization, A has the higher proportion of patients reporting quality as good (rather than OK or poor) but a lower proportion reporting quality as either good or OK (rather than poor). It follows that neither approach can provide a robust basis for an analysis of the variation in quality between practices.

The calculation of the lottery index may be thought of in terms of the outcome of a lottery in which the patient has an equal chance of being assigned to A or B with the quality level for each practice determined by a random draw from the quality profile for that practice. The patient “wins” or “loses” depending on whether they are assigned to the practice with the higher or lower randomly chosen quality level, and will be indifferent to the lottery outcome if the quality levels delivered by the two practices are the same. Patients have a (15 + 15 + 3) = 33% chance of “winning” if assigned to A, a (4 + 16 + 12) = 32% chance of “winning” if assigned to B and will be indifferent to the lottery outcome in the remaining (20 + 3 + 12) = 35% of draws. Hence the difference in “winning” chances of (33 − 32) = 1% provides a measure of the degree to which the profile of A is superior to that of B. We proceed to calculate the lottery index as the absolute value of this difference, where this is equal by definition to the absolute difference in the chances that a patient randomly assigned to one practice will receive better rather than worse care than if assigned to the other.

More generally, consider some population in which each individual is a patient of one (and only one) of a set of *K* ≥ 2 healthcare providers, such that the patient list of each provider is independent of that of any other. Let $P\left(Q_{k}\geq Q_{k^{\prime }}\right)=P\left(Q_{k}>Q_{k^{\prime }}\right)+P\left(Q_{k}=Q_{k^{\prime }}\right)$ be the probability that the quality of care received by a randomly chosen patient with provider *k* ∈ *K* is at least as good as—that is, strictly better than or the same as—that received by a randomly chosen patient with provider *k*′ ∈ *K*. Following Allanson (2017, 2021), the *pairwise quality difference* is defined as the difference in chances that the quality of care received by a randomly chosen patient with provider *k′* is (strictly) better rather than worse than that received by one with provider *k*:

(1) $$\Delta_{kk^{\prime }}=-\Delta_{k^{\prime }k}=P\left(Q_{k^{\prime }}\geq Q_{k}\right)-P\left(Q_{k}\geq Q_{k^{\prime }}\right)=P\left(Q_{k^{\prime }}>Q_{k}\right)-P\left(Q_{k}>Q_{k^{\prime }}\right);\;\forall k,k^{\prime }\in K$$

$\Delta_{kk^{\prime }}$ will take a value of zero if the quality profiles of the two providers are equivalent,^1^ although this does not necessarily imply that they are identical; a maximum value of one when the worst quality of care provided by provider *k′* is strictly better than the best quality provided by provider *k*; and a minimum value of minus one when the opposite is the case.

The normative significance of the pairwise quality difference derives from the use of the statistical preference criterion (De Schuymer et al., 2003) for the comparative evaluation of quality profiles. According to this criterion one profile is better than another if the patient receiving the (strictly) higher quality care of any randomly chosen pair of patients is more likely to be registered with the first rather than the second provider. The criterion is more general and powerful than first‐order stochastic or rank dominance (De Baets & De Meyer, 2007), which is commonly employed to compare ordinal distributions but can lead to incomplete orderings (see e.g., Gutacker & Street, 2018). Statistical preference will always say whether one quality profile is better, worse or equivalent to another, whereas rank dominance often leaves things undefined—neither better nor worse, but not equivalent either. Thus, A and B in the numerical example are not comparable by rank dominance since the proportion of patients who receive poor care is lower in B but the proportion receiving no better than OK care is lower in A. Moreover, statistical preference is not only able to rank all quality profiles but also provides a “graded” comparison of them (De Baets & De Meyer, 2007), with the pairwise difference in winning chances offering a readily intelligible measure of the degree to which one profile is better or worse than another.

### 2.2 The comparative quality index

In the absence of an external standard, a summary measure of comparative quality for each provider can be obtained by calculating a pairwise index for it relative to some common benchmark patient quality profile, such as that of the whole population (Allanson, 2021). The *comparative quality index*:

(2) $$\Delta_{k}=\sum_{k^{\prime }=1}^{K}p_{k^{\prime }}\left(P\left(Q_{k}>Q_{k^{\prime }}\right)-P\left(Q_{k^{\prime }}>Q_{k}\right)\right)=\sum_{k^{\prime }=1}^{K}p_{k^{\prime }}\Delta_{k^{\prime }k};\;\forall k\in K$$

offers a summary measure of the quality of provider *k* compared to all *K* providers, where $p_{k^{\prime }}$ is the proportion of total registrations with provider *k′*. The index may be used to generate a complete ranking of providers by quality but will generally be more informative than a simple measure of “league table” position. Δ_*k*_ can take values in the closed interval from –(1 – *p*_*k*_) to +(1 – *p*_*k*_), since Δ_*kk*_ = 0 by definition, with the sign of the index indicating whether the care quality of provider *k* is better or worse than the benchmark and its magnitude indicating the degree of any separation between the two profiles. By construction, Δ_*k*_ takes a weighted average of zero across all providers, that is, ∑_*k*_ *p*_*k*_Δ_*k*_ = 0.

### 2.3 The lottery index

The *lottery index* provides a measure of the variation in quality between providers in terms of the average absolute value of the pairwise quality differences over all pairs of providers (Allanson, 2021). Specifically, the index is defined as the normalized average absolute value:

(3) $$L=\left(\sum_{k=1}^{K}\sum_{k^{\prime }=1}^{K}p_{k}p_{k^{\prime }}\left|\Delta_{kk^{\prime }}\right|\right)/\left(1-\sum_{k=1}^{K}p_{k}^{2}\right)$$

where the normalization factor $\left(1-\sum_{k}p_{k}^{2}\right)$ implies that *L* may be interpreted as the patient‐weighted mean absolute difference in the chances that quality will be better rather than worse as a result of being cared for by one provider rather than another. The interpretation in terms of the average absolute difference in the chances of winning rather than losing over all distinct pairwise lotteries follows directly from the definition of the pairwise index $\left|\Delta_{kk^{\prime }}\right|$.

Alternatively, the index may be interpreted as a measure of the potential value to patients of exercising the right to choose their healthcare provider rather than it being determined by the accident of where they live. This follows since $\left|\Delta_{kk^{\prime }}\right|$ in Equation (3) may also be written as:

(4) $$\left|\Delta_{kk^{\prime }}\right|=2\max\left\{P\left(Q_{k^{\prime }}>Q_{k}\right),P\left(Q_{k}>Q_{k^{\prime }}\right)\right\}-\left(P\left(Q_{k^{\prime }}>Q_{k}\right)+P\left(Q_{k}>Q_{k^{\prime }}\right)\right);\;\forall k,k^{\prime }\in K$$

So *L* may also be interpreted as twice the mean increase in the probability that patient care will be better than it would otherwise have been if patients chose the provider with the better quality profile of any pair of providers rather than being randomly assigned to one of them.

A third interpretation is in terms of the degree of “postcode discrimination” faced by patients on the basis of where they live due to the variation in care quality across providers. Specifically, *L* may be interpreted as a summary measure of discrimination between pairs of providers given that $\Delta_{kk^{\prime }}$ is formally equivalent to the Le Breton et al. (2008) first‐order discrimination index Δ_1_ if provider *k′* has the better profile of the two providers.

*L* will take a minimum value of zero if and only if the comparative quality—but not necessarily the quality profiles—of all providers is the same and a maximum value of one if there is complete separation of the patient lists for each provider into disjoint strata in the population quality profile. The index is sensitive to any change in the quality of care received by any patient unless the change is over some quality range occupied exclusively by others cared for by the same provider as the patient. For binary 0/1 quality indicators (e.g., good or bad), *L* is simply the weighted average of the absolute pairwise differences in the proportion of patients receiving good care. But, as shown by the example, it can also be calculated for ordinal measures with three or more categories without the need for dichotomization.

Given independent patient lists, the simplest way to compute *L* for an ordinal quality indicator is to calculate the pairwise indices using the approach employed in the numerical example and then take the weighted average over all pairs. A more computationally efficient approach if there are more than three health categories makes use of the relation $\Delta_{kk^{\prime }}=\left(1-2\left[P\left(Q_{k}>Q_{k^{\prime }}\right)+0.5P\left(Q_{k}=Q_{k^{\prime }}\right)\right]\right)$ in the first step. Supporting Information S1: Appendix 1 provides Stata code to compute values of $\Delta_{kk^{\prime }}$, Δ_*k*_ and *L* for a set of healthcare providers from ordinal quality data. For cardinal indicators, the pairwise indices can be calculated exactly from the relation $\Delta_{kk^{\prime }}=G_{b}/G_{B}$ if practice *k′* has the higher mean quality of the two providers (Monti & Santori, 2011), where *G*_*b*_ is the conventional between‐group Gini coefficient (Pyatt, 1976) and *G*_*b*_ and is the variant proposed in Yitzhaki and Lerman (1991). Alternatively, *L* may be approximated to any required degree of accuracy by rounding the data and then treating the resultant discretized variable like any other ordinal indicator. For both types of indicator, the measures are calculated directly from the data not from predicted or simulated quality profiles (see e.g., Gutacker & Street, 2018).

### 2.4 Standardization of practice quality profiles

Previous studies have revealed systematic differences in how patients from different socio‐demographic groups evaluate the quality of primary care services (see e.g., Lyratzopoulos et al., 2012; Paddison et al., 2012). Individual response data from the GPPS could in principle be used to estimate directly standardized quality profiles calculated on the basis that all practices had the same socio‐demographic composition as the whole population. However, the sample size of the GPPS is not large enough to provide reliable estimates of group‐specific quality profiles at the practice level and the approach is in any case inapplicable to the practice‐wide CQC ratings and QOF scores. Instead we employ an indirect standardization procedure based on the estimation of a distribution regression model (Chernozhukov et al., 2013) for each quality indicator to predict the practice quality profiles that would be expected if quality outcomes conditional upon socio‐demographic characteristics were the same in each practice as in England as a whole. Specifically, the proportion of the patients of a practice expected to experience a quality level no better than *q* (*q* = 1, … Q − 1 of Q discrete quality levels) is given by the prediction from a binary choice model in which the dependent variable takes a value equal to the proportion of patients reporting experience no better than *q*.

## 3 DATA AND METHODS

### 3.1 Data

Patient experience data for 6926 practices were obtained from the 2019 results of the annual GPPS (NHS England, 2019). The survey asked patients about a range of issues associated with using the services offered by their practice, including how they would describe their overall experience using a 5‐category semantic differential scale, as well as various questions about their own personal circumstances. The specific question was: “Overall, how would you describe your experience of your GP practice?”, with response categories: “Very good”, “Fairly good”, “Neither good nor poor”, “Fairly poor”, “Very poor”. Postal questionnaires were sent out in January 2019 to 2.33 million adult patients in England of whom 770512 in 6999 practices completed the survey representing a response rate of 33.1% (Ipsos MORI, 2019). All practices listed on NHS Digital as having eligible patients were included in the survey apart from an unspecified number that chose to opt out as they felt it was inappropriate to their patient population. Patients were eligible for inclusion in the survey if they had a valid NHS number, had been registered with a practice continuously for at least 6 months before being selected, and were 16 years of age or over. The sample was based on a proportionately stratified, unclustered design, with the sample size for each practice selected to ensure that confidence intervals were as consistent as possible between practices. Practice‐level data are published on a weighted basis to ensure that the results are more representative of the population of adult patients registered with each practice by correcting for the sampling design and to reduce the impact of non‐response bias. No overall experience data are available for 73 practices due to the suppression of data for questions answered by fewer than 10 people to protect confidentiality.

Inspection ratings data for 6670 practices was obtained from the January 2019 CQC Care Directory (CQC, 2019a). The Care Directory is updated monthly and includes the latest published ratings of all practices that have been subject to inspection in England, which in January 2019 dated back as far as November 2014. Practices are given an overall rating for the “whole population” of service users on a 4‐category semantic differential scale following a visit by an inspection team and taking account of the views of both patients and staff. The overall rating is based on a detailed assessment of the quality of care across six patient subgroups in terms of whether the service is safe, effective, caring, responsive to people's needs and well‐led. The CQC uses a risk‐based approach to target inspections in which practices rated “Inadequate” and “Requiring improvement” are required to make changes and subject to re‐inspection within six and 12 months respectively, while those rated “Good” or “Outstanding” are not liable to re‐inspection unless there is monitoring evidence of quality change (CQC, n.d.). The most recent rating was used for practices with multiple ratings based on different inspection dates. The rating for the main branch of a practice was used where ratings were available for more than one location.

QOF scores for 6854 practices with achievement data were obtained from the QOF 2018‐19 results (NHS Digital, 2019). The QOF is a voluntary, annual incentive payment scheme for all practices in England that rewards practices for the provision of “quality care”, with 95.1% of practices participating in the reporting year from April 1, 2018 to March 31, 2019. The QOF provides an indication of overall practice achievement through a points system, with points awarded against a range of 77 clinical care and public health indicators based, for example, on the proportion of patients on specified disease registers who receive defined interventions. The headline measure of practice achievement published by NHS England is percentage attainment of the maximum 559 QOF points available, but an alternative measure is also provided which takes account of instances where practices cannot achieve points because they have no patients pertinent to an indicator. We use the publicly reported scores and refrain from making an adjustment by adding “exception reported” patients back into the population denominator, which typically provides a less favorable measure of performance. QOF percentage attainment data are rounded to 1 decimal place to calculate the indirectly standardized quality profiles.

### 3.2 Methods

The main analysis of patient experience data was based on the full GPPS sample of 6926 practices. A sub‐set of 6427 matched practices with valid GPPS, CQC and QOF data was used to generate comparable CCG‐level results for all three practice quality indicators. All sample practices belonged to one of 195 CCGs, with the number per CCG varying between 10 and 169, and a mean of 35.5. Practice weights based on the number of registered patients aged 16 years old and over in December 2018 (NHS Digital, 2018) were used to construct CCG quality profiles as weighted averages of sample practice profiles and, after adjusting for missing practices within each CCG, to ensure the national representativeness of results at the CCG level.

Practice‐level comparative quality and within‐CCG lottery indices were calculated using the GPPS practice quality profiles, and CCG‐level comparative quality and between‐CCG lottery indices using the CCG quality profiles for all three indicators. Analysis of the CQC and QOF data was restricted to the CCG level because the practice‐level quality profiles for these indicators are degenerate distributions, consisting simply of an overall rating or score. This does not prevent the application of the measurement framework at the practice level but it does limit the informational value of the resultant indices. In particular, for continuous quality indicators the comparative quality indices of practices will simply be given by their rank in the population‐level quality distribution less half, while the between‐practice lottery index will equal one in the absence of ties.

We report both total and indirectly standardized indices. For the estimation of indirectly standardized quality profiles, distribution regression models for each practice quality measure were specified as a function of sex, age, ethnicity, health and deprivation, where the models allow for main effects only given the nature of the socio‐demographic data. The composition of each practice patient list in terms of sex (female, male), age group (16–24, 25–34, 35–44, 45–54, 65–74, 75–84, 85+), ethnicity (White, Asian, Black, Mixed, Other) and health status (presence of long‐term condition) are separately reported in the GPPS data. One missing health status value was replaced by the CCG mean for the practice. Deprivation was measured by the 2019 Index of Multiple Deprivation score for the Lower Super Output Area in which the practice was located (Ministry of Housing, Communities & Local Government, 2019). The specifications also include a set of intercept dummy variables to allow for separate impacts on practice quality attributable to CCGs themselves. Predictions for each practice were based on the socio‐demographic characteristics of the practice and CCG shares of the English patient population to avoid adjusting for factors over which CCGs may have influence. In our base case analysis we employ a linear probability distribution regression model (LPDRM) for convenience but, as a robustness check, also calculate indirectly standardized profiles using a generalized linear distribution regression model (GLDRM) with a probit link function and a binomial distribution with the parameter *n* set equal to the number of survey responses in a practice for the GGPS data, and to one for the CQC and QOF data. Estimated counterfactual cumulative proportions were censored where necessary to lie in the unit interval, with the resultant set of predictions scaled to match the sample mean. Finally, bootstrap standard errors were obtained for all comparative quality and lottery indices by the resampling of practices within each CCG to reflect the organizational structure. All analysis was conducted using Stata version 15.1.

## 4 RESULTS

We first present results based on the full sample of practices with GPPS patient experience data, looking in turn at the indices calculated using the practice and CCG‐level quality profiles. We subsequently compare the indices calculated using the GPPS, CQC and QOF CCG‐level quality profiles constructed from the matched sample of practices with valid data for all three indicators.

### 4.1 GPPS patient experience

This section reports results based on the full GPPS sample of 6926 practices. The proportions of adult patients in England reporting their overall experience of their practice as very poor, fairly poor, neither good nor poor, fairly good and very good were 2.1%, 4.4%, 10.6%, 37.8% and 45.1% respectively. Scoring these responses 1–5, practice quality was 4.19 on average with a standard deviation of 0.30 across all practices. It might thus appear that the variation in reported experience between practices was low relative to the mean, but the coefficient of variation can be made arbitrarily large or small through the choice of alternative scoring schemes. For example, if the responses were scored instead from −2 to +2, with 0 providing a natural measure of neither good nor poor, then the coefficient of variation would be 24.9% not 7.1%. Some other approach is therefore required to meaningfully assess the degree of variation in reported experience.

**FIGURE 2:**
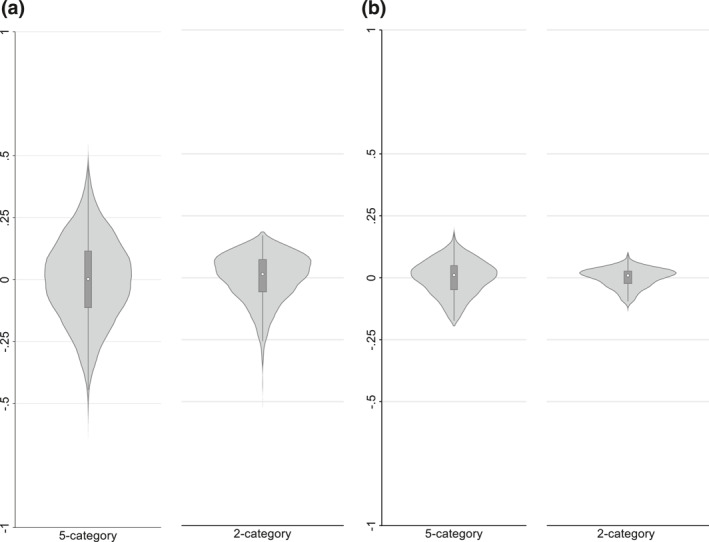
Violin plots of comparative GP patient survey patient experience indices for the whole of England. (a) Practices (b) clinical commissioning groups

Figure 2a shows the distribution of practice‐level comparative quality index values, which have a patient‐weighted mean of zero by construction. The variation in comparative quality across individual practices is considerable, ranging from a 0.520 or 52.0 percentage point (pp) higher chance that a patient from the best practice would have reported a better rather than worse experience than one from anywhere in England to a 61.4pp lower chance for the worst practice. The standard deviation of the comparative quality index is 16.8pp, with within‐CCG differences accounting for 83.0% of the variance in practice‐level comparative quality and only 17.0% due to between‐CCG differences. Thus there was much more variation between practices within each CCG than between CCGs, where the former is of more relevance for the exercise of patient choice given the evidence that patients are only willing to travel a limited distance to access better quality GP services (Santos et al., 2017).

Responses to the patient experience question are commonly collapsed into a dichotomous variable for presentational purpose by combining very poor/fairly poor/neither good nor poor into one category and fairly good/very good into the other (see e.g., NHS England and Ipsos MORI, 2019). However, the use of this binary quality indicator leads to a marked reduction in the ability to discriminate between “average” and “good” practices, while continuing to capture the extent to which “bad” practices offer poorer quality care. Thus, a patient from the best practice is now estimated to have had only a 17.1pp higher chance of reporting a better rather than worse experience than one from anywhere in England, whereas a patient from the worst practice would have had a 50.7pp lower chance. Overall, dichotomization leads to a substantial underestimate of the variation in the quality of care between practices with the standard deviation of the comparative quality index falling to 9.8pp as a result.

**FIGURE 3:**
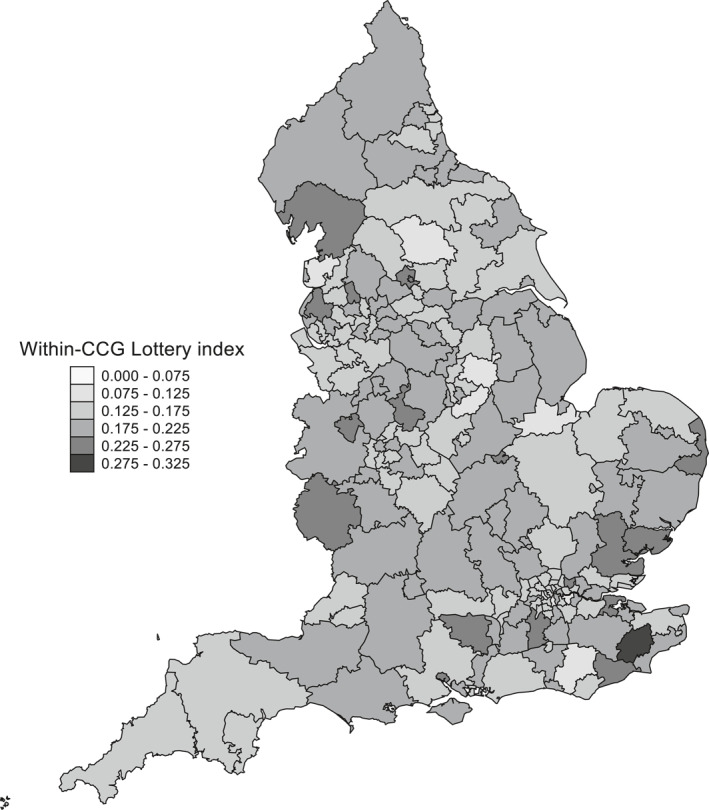
Variation in quality between practices within individual clinical commissioning groups. Figure based on GP patient survey 5‐category patient experience responses

The first row ([a] Full sample GPPS) of results in Table 1 reports an average 17.8pp absolute difference in the chances that patient experience was better rather than worse as a result of being registered with one practice rather than another within the same CCG. Thus, on average, it was 8.9pp (=17.8/2) more likely that patient experience would have been better than it would otherwise have been as a result of being able to choose the better of any pair of practices within a CCG rather than being randomly assigned to one of them. Figure 3 maps the variation in quality between practices within individual CCGs, ranging from a 9.0pp absolute difference in patients' chances of reporting a better rather than worse experience as a result of being registered with one practice rather than another in the most homogeneous CCG to a 30.3pp difference in the least. The expected value of the within‐CCG lottery index is not a function of the number of practices within a CCG although, unsurprisingly, the conditional variance is decreasing in the number of practices. Moreover, differences between the socio‐demographic composition of practices within individual CCGs account for relatively little of the total variation in practice quality within CCGs, with predicted within‐CCG variation highest in the more heterogeneous and segregated metropolitan areas based on the distribution regression estimates in Supporting Information S1, Tables A2.1 and A2.2. Table 1 reports that the within‐CCG lottery index based on the LPDRM indirectly standardized profile was 5.9pp rather than 17.8pp, leaving a residual or “unexplained” 11.9pp average absolute difference in the chances that reported patient experience would have been better rather than worse as a result of being registered with one practice rather than another within the same CCG.

**TABLE 1:** Lottery indices

	Raw or unadjusted	LPDRM results		GLDRM results	
		Indirectly standardized	Residual	Indirectly standardized	Residual
(a) Full sample GPPS					
Average within‐CCG indices					
GPPS 5‐category	0.1775**	0.0589**	0.1186**	0.0610**	0.1165**
	*0.0015*	*0.0023*	*0.0026*	*0.0023*	*0.0026*
GPPS 2‐category	01002**	0.0332**	0.0670**	0.0337**	0.0665**
	*0.0010*	*0.0013*	*0.0018*	*0.0014*	*0.0018*
Between‐CCG indices					
GPPS 5‐category	0.0789**	0.0520**	0.0269**	0.0526**	0.0263**
	*0.0027*	*0.0025*	*0.0039*	*0.0024*	*0.0038*
GPPS 2‐category	0.0433**	0.0288**	0.0145**	0.0283**	0.0150**
	*0.0016*	*0.0015*	*0.0025*	*0.0015*	*0.0025*
(b) Common sample					
Between‐CCG indices					
GPPS 5‐category	0.0789**	0.0512**	0.0274**	0.0517**	0.0272**
	*0.0024*	*0.0024*	*0.0031*	*0.0028*	*0.0032*
CQC 4‐category	0.0986**	0.0184**	0.0802**	0.0033**	0.0953**
	*0.0064*	*0.0033*	*0.0072*	*0.0005*	*0.0065*
QOF cardinal (% achievement 559)	0.2931**	*∼*	*∼*	*∼*	*∼*
	*0.0088*				
Clinical domain	0.2875**	*∼*	*∼*	*∼*	*∼*
	*0.0084*				
Public health domain	0.2259**	*∼*	*∼*	*∼*	*∼*
	*0.0087*				
Public health AS domain	0.2313**	*∼*	*∼*	*∼*	*∼*
	*0.0077*				
QOF cardinal (% achievement practice max)	0.2931**	*∼*	*∼*	*∼*	*∼*
	*0.0088*				
QOF discretized	0.2925**	0.0899**	0.2026**	0.1044**	0.1880**
	*0.0091*	*0.0090*	*0.0137*	*0.0115*	*0.0135*
QOF 5‐category	0.2577**	0.0811**	0.1766**	0.0888**	0.1689**
	*0.0068*	*0.0097*	*0.0129*	*0.0093*	*0.0103*

*Note*: ‘Residual’ indices are calculated as the difference between the corresponding raw and indirectly standardized indices and reflect that part of the total variation in care quality not ‘explained’ by the relevant distribution regression model. Bootstrapped standard errors based on 50 replications are in italics.

*p < 0.05, **p < 0.01.

*Source*: Own calculations from GPPS, CQC and QOF data.

Figure 2b shows that there was also considerable variation in the comparative quality levels of CCGs, ranging from a 18.3pp higher chance that a patient from the best CCG would have reported better rather than worse experience than one from anywhere in England to a 17.4pp lower chance for the worst CCG. Dichotomization again leads to a reduction in measured variation, particularly between “average” and “good” CCGs: the range in chances shrinks to 9.3pp higher for the best CCG to 12.6pp lower for the worst, that is to the difference in the proportion of patients reporting their experience as either fairly or very good between the best and worst performing CCGs (cf. NHS England and Ipsos MORI, 2019, p. 10). Dichotomization also leads to some re‐ranking of CCGs in terms of performance, with the Kendall's rank correlation coefficient *τ*_a_ between the two rankings implying that the full and dichotomized measures are 86.5pp (95% CI, 0.837–0.893) more likely to agree than differ over which of any pair of CCGs had the strictly better quality profile (cf. Newson, 2002).

**FIGURE 4:**
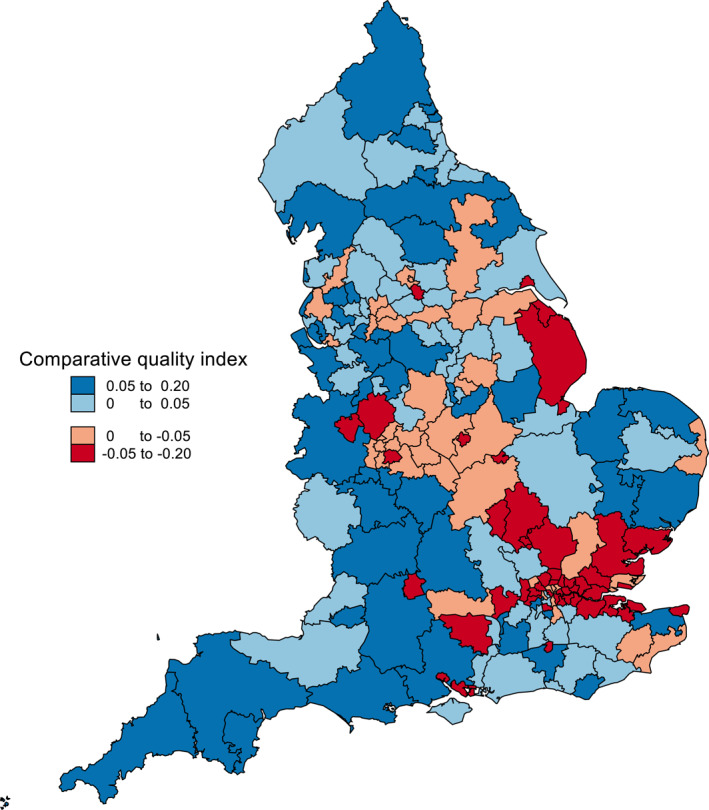
Comparative quality of clinical commissioning groups. Figure based on GP patient survey 5‐category patient experience responses

Figure 4 maps the comparative quality of CCGs and shows that patient experience tends to be worse in CCGs located in metropolitan regions and surrounding areas than in the more rural “shire” counties. This geographical pattern is strongly associated with socio‐demographic differences between CCGs, with the LPDRM estimates in Supporting Information S1: Table A2.1 implying that patient experience would be expected to have been worse in CCGs containing higher proportions of patients of prime working age (25–54 year olds), in the Asian ethnic group, with long‐term health conditions and living in more deprived areas. The between‐CCG lottery index would have been 5.2pp rather than 7.9pp if the only source of variation in practice quality was differences in the socio‐demographic composition of patient lists, leaving an unexplained or residual 2.7pp absolute difference in the chances that patient experience was better rather than worse as a result of being registered with one CCG rather than another. Dichotomization leads to a loss of contrast between better and worse performing CCGs but no fundamental change in the geographical pattern.

### 4.2 Comparative analysis of three practice quality measures

**FIGURE 5:**
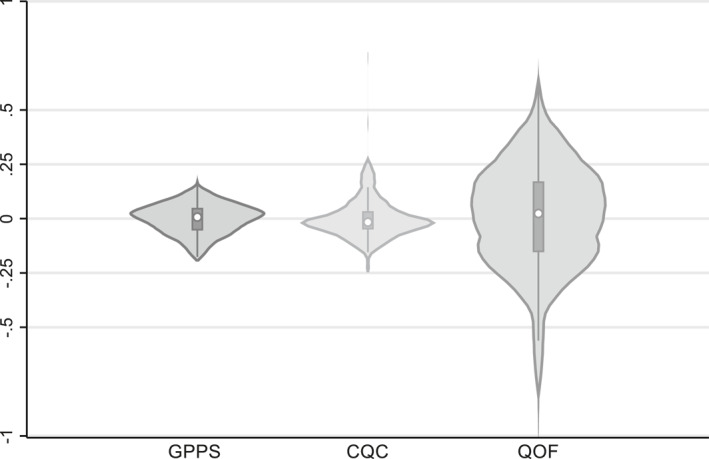
Violin plots of raw clinical commissioning group comparative quality indices based on alternative measures

This section reports results based on the matched sample of 6427 practices with valid GPPS, CQC and QOF quality data. The left‐hand plot in Figure 5 and first row ([b] Common sample) of Table 1 present results based on the GPPS CCG‐level quality profiles, which are virtually the same as those discussed above for the full GPPS sample. We compare these results to those obtained with the CQC and QOF indicators.

The proportions of the patient population in England registered at a practice with a latest CQC rating of inadequate, requires improvement, good and outstanding were 0.7%, 2.9%, 90.4% and 6.0% respectively. This profile is likely to exaggerate the quality of GP services in January 2019 to the extent that the CQC inspection regime was quicker at picking up improvement in poorly rated practices than deterioration in highly rated ones. But limiting the analysis to practices that have been recently inspected may lead to the opposite problem as poorer quality practices were targeted for re‐inspection.^2^ Figure 5 plots CCG comparative quality indices based on the latest CQC inspection ratings of all practices, ranging from a 74.7pp higher chance that a patient from the best CCG would have been in a practice with a higher rather than lower rating than one from anywhere in England to a 22.8pp lower chance for the worst CCG. However the best CCG—comprising a few, mostly outstanding practices—is an extreme outlier and the between‐CCG lottery index of 9.9pp reported in Table 1 is not that much higher than that of the GPPS measure despite the much larger range.

The association between the ranking of CCGs by CQC inspection rating and GPPS patient experience is positive but weak. Kendall's *τ*_a_ is only 0.305 (95% CI, 0.203–0.406), implying that there was only a 30.5pp higher chance that the two measures would agree rather than differ over which of any pair of CCGs had the strictly better quality profile. The null hypothesis that *τ*_a_ is equal to 1, which would be the value if the two measures produced identical rankings of CCGs, can be rejected decisively implying that GPPS patient experience and CQC inspection rating data do not provide alternative sources of ordinally equivalent information on some common latent “primary care quality” characteristic. Unlike for the GPPS measure, very little of the variation in inspection ratings between CCGs can be accounted for by practice‐level differences in socio‐demographic composition. The distribution regression results are given in Supporting Information S1 Tables A2.3 and A2.4, with Table 1b reporting an LPDRM indirectly standardized lottery index of 0.0184 that is only 18.6% of the raw value.

Levels of QOF achievement were very high with 14.5% of patients registered in practices achieving the maximum score of 559 QOF points, mean percentage achievement of 96.9pp (541.6 points), and standard deviations of 5.4pp (30.2 points) and 3.0pp (16.7 points) at the practice and CCG levels respectively. The right hand plot of CCG comparative quality indices in Figure 5 is based on QOF scores, ranging from a 66.2pp higher chance that a patient from the best CCG would have been in a practice with a higher rather than lower QOF score than one from anywhere in England to a 93.3pp lower chance for the worst CCG. Table 1b reports a between‐CCG lottery index of 0.2931 based on percentage achievement of the maximum score, with the alternative measure of percentage achievement of points available to the practice yielding the same result to 4 significant figures. Lottery indices for the separate clinical, public health and public health additional services domains are somewhat lower, but all are above 0.2 despite more than half of practices achieving the maximum score in the latter two domains.

These considerably higher estimates of the variation in care quality compared to both the GPPS and CQC indices cannot simply be dismissed as an artifact of the cardinality of QOF scores: collapsing the total QOF score into a 5‐category variable with population proportions for England as a whole identical to those for the GPPS measure only reduces the index value to 0.2577. Rather they would appear to reflect the relatively high degree of variation in QOF scores between CCGs as compared to within CCGs, with the between‐CCG standard deviation of 3.0pp reported above similar in magnitude to a weighted‐average within‐CCG standard deviation of practice quality of 3.8pp: between‐CCG differences accounted for as much as 30.4% of the overall variance in practice‐level total QOF scores.

The associations between the ranking of CCGs by QOF achievement and by the other two quality indicators are both weakly positive, with Kendall's *τ*_a_ equal to 0.333 (95% CI, 0.236–0.431) for GPPS patient experience and 0.267 (95% CI, 0.160–0.374) for CQC inspection ratings. Only 30.7% of variation (0.0899/0.2925) in QOF achievement between CCGs was accounted for by differences in the socio‐demographic composition of practice lists, with the GLDRM yielding a somewhat higher estimate of the proportion of “explained” variation in this case. Illustrative distribution regression model results for QOF achievement are presented in Supporting Information S1 Tables A2.5 and A2.6.

**FIGURE 6:**
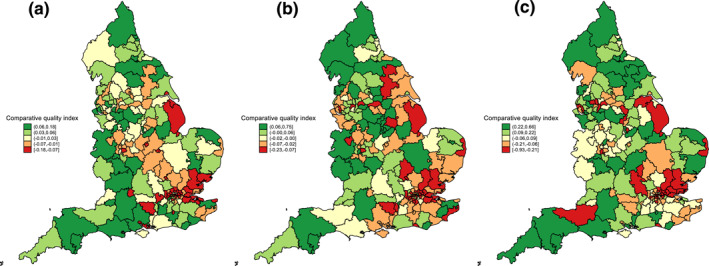
Comparative quality indices by clinical commissioning group quintile based on alternative primary care quality indicators: (a) GP patient survey 5‐category indicator (b) care quality commission inspection ratings (c) quality and outcomes framework achievement scores

By way of summary, Figure 6 maps the comparative quality indices by CCG quintile for the three alternative practice quality indicators. The maps share some similar features, which is to be expected given the positive association between the corresponding comparative quality indices. In particular, all show a concentration of CCGs with poorer levels of primary care quality in the London area. Nevertheless, the prevailing impression is of pervasive differences in the ranking of individual CCGs across the three measures, with nine CCGs in the top quintile on one measure and the bottom on another.

## 5 DISCUSSION

Evidence on the quality of healthcare services is increasingly being provided by multicategory response information from patient experience surveys, supplementing the routine collection of standard cardinal quality indicators. This paper proposes an assessment framework that is directly applicable to both ordinal and cardinal quality indicators, providing intelligible measures of both the comparative quality of each member of a set of healthcare providers serving some population and the variation in quality between them.

Our approach is motivated by the concept of statistical preference whereby one healthcare provider is judged to be better than another if the patient receiving the (strictly) higher quality care of any randomly chosen pair of patients is more likely to be registered with the first rather than the second provider. Unlike first order stochastic dominance, statistical preference will provide a graded comparison of all possible pairs of care quality profiles. The resultant measures are sensitive to the full distribution of quality scores for each provider, not just the mean nor the proportion meeting some binary quality threshold.

The GPPS offers a large‐scale, annual survey of patients' experience in virtually all practices in England, with practice‐level multicategory response data made publicly available in a timely fashion. We find significant variation in primary care quality levels both between practices within individual CCGs and between CCGs in 2019, with the right to choose between any two practices within a CCG leading on average to an 8.9pp higher chance that patient experience would be better than it would otherwise have been under random assignment. Dichotomization leads to a reduction in measured variation, with the loss of contrast most marked between “average” and “good” providers. Practice‐level information on primary care quality is also available in the form of ordinal CQC inspection ratings and cardinal QOF achievement scores, which are generated for regulatory and performance incentive purposes respectively. We show that neither provide an alternative source of ordinally equivalent information on some common latent “primary care quality” variable to either the GPPS or each other. Allen et al. (2020) have previously found a similar lack of agreement between routine performance indicators, measuring patient satisfaction and the management of chronic conditions, and CQC inspection ratings based on the limited ability of the former to predict the latter. Additionally, the measured level of between‐CCG variation is much higher using QOF scores than with the other two quality indicators. Why this is the case is unclear though we do demonstrate that it is not due to the cardinality of the QOF indicator by showing that the value of the lottery index is relatively insensitive to the grouping of QOF scores.

Elimination of the postcode lottery in GP patient experience would provide a measurable, policy‐relevant objective to the extent that such variation was due to factors within the control of the National Health Service. In particular, attainment of the goal would not require that all individual patients could expect to receive the same quality of care, which is surely unrealistic, but rather that their experience was equally likely to be better rather than worse as a result of being registered with one practice or CCG rather than another to the extent that this was achievable. A limitation of our study is that the indirect standardization procedure is based on practice‐level rather than individual patient data, which prevents the specification of interaction terms between socio‐demographic characteristics and runs the risk of ecological bias. Nevertheless our findings are consistent with those from other studies (see e.g., Lyratzopoulos et al., 2012; Paddison et al., 2012) in showing that patient experience tended to be worse in practices located in more deprived areas with higher proportions of patients of prime working age, in the Asian ethnic group and in poorer health. These variations may be due to differences in reporting behavior between different patient subgroups and/or systematic disparities in the actual standard of care provided to them, with our finding that socio‐demographic characteristics explained much smaller but still significant proportions of between‐CCG variation in both CQC ratings and QOF scores suggesting that both factors were of importance (see e.g., Lyratzopoulos et al., 2012, Burt at al., 2016; Fisher et al., 2020; for further evidence on this point).

In conclusion, the proposed approach provides a general framework to assess variation between healthcare providers or geographical areas making full use of the information provided by the ordinal quality indicators that are now routinely available. Further studies are required both to elicit healthcare service decision makers' views on the utility of our proposed new performance metric and to explore whether our empirical findings are more generally characteristic of the scale of healthcare variation in other clinical settings and countries. Finally, we note that the statistical preference criterion can also be used to compare care quality profiles that are not independent of each other. In particular it would be of interest to evaluate changes in healthcare provider quality over time taking account of the temporal dependence of patient experience, with the impact of the COVID‐19 epidemic on GP practice quality an obvious topic for investigation.

## CONFLICT OF INTEREST

No conflicts of interest exist.

## ETHICS STATEMENT

The paper raises no ethical issues.

## Supporting information

Supporting Information S1

## Data Availability

The data that support the findings of this study were derived from the following resources available in the public domain: CQC: Care Directory with ratings (January 02, 2019). https://www.cqc.org.uk/about‐us/transparency/using‐cqc‐data Ministry of Housing, Communities & Local Government: English indices of deprivation 2019. https://www.gov.uk/government/statistics/english‐indices‐of‐deprivation‐2019 NHS Digital: Patients registered at a GP Practice ‐ December 2018. https://digital.nhs.uk/data‐and‐information/publications/statistical/patients‐registered‐at‐a‐gp‐practice/december‐2018 NHS Digital: Quality and Outcomes Framework, Achievement, prevalence and exceptions data 2018‐19 [PAS]. https://digital.nhs.uk/data‐and‐information/publications/statistical/quality‐and‐outcomes‐framework‐achievement‐prevalence‐and‐exceptions‐data NHS England: The GP Patient Survey: Practice report (2019 publication). Available at: https://gp‐patient.co.uk/surveysandreports2019.

## ENDNOTES

^1^
It may be noted that $\Delta_{kk^{\prime }}=0$ implies $\Theta_{kk^{\prime }}=P\left(Q_{k^{\prime }}>Q_{k}\right)/P\left(Q_{k}>Q_{k^{\prime }}\right)=1$, where $\Theta_{kk^{\prime }}$ may be interpreted as an odds ratio if the quality indicator is a binary variable but not otherwise.

^2^
For example, the proportion of patients registered at practices with published ratings from 2018 onwards was 3.9%, 13.9%, 79.3% and 3.0%.
