# Supplementary material for: Comparing healthcare quality: A common framework for both ordinal and cardinal data with an application to primary care variation in England
Source: Health Econ. 2022 Aug 28;31(12):2593–608. doi: 10.1002/hec.4597 (PMC9804671; doi:10.1002/hec.4597)
Supplement: Supplementary file 1 — Supporting Information S1 [file HEC-31-2593-s001.docx]

**Appendix 1. Stata code for computing pairwise quality differences and summary indices**

This code will compute pairwise quality differences, comparative quality indices and the lottery index for a set of healthcare providers from multi-category ordinal quality profile data stored in an Excel file quality_profile_data.xlsx. It is assumed that the variables names are given in the first row of the file and each subsequent row holds all of the data on one healthcare provider, consisting of an identifier (hpid); a provider weight (hpw), where the weights sum to one across providers; and a set of variables giving the proportions of patients in each of *C* quality categories (qpfreq1, qpfreq2, … qpfreqC), which sum to one across categories.

import excel using quality_profile_data.xlsx, firstrow clear

global hpnum=_N

reshape long qpfreq, i(hpid) j(qual)

* set missing values (if any) to zero

replace qpfreq=0 if qpfreq==.

* Calculate "average" cumulative frequencies

sort hpid qual

by hpid: gen qofreq=sum(qpfreq)-0.5*qpfreq

* create matrices from variables

reshape wide qpfreq qofreq, i(hpid) j(qual)

mkmat hpw, matrix(matwgt)

mkmat qpfreq*, matrix(matqpf)

mkmat qofreq*, matrix(matqof)

** Calculation of pairwise quality differences: mrcpqd[$hpnum,$hpnum]

matrix mat1 = matqpf*matqof'

matrix mat1sqr=J($hpnum,$hpnum,1)

matrix mrcpqd = mat1sqr-2*mat1

matrix list mrcpqd

** Calculation of comparative quality: mhpcompq[1,$hpnum]

matrix mhpcompq = matwgt'*mrcpqd

matrix list mhpcompq

* check that comparative quality indices sum to zero

matrix msumcheck = mhpcompq*matwgt

matrix list msumcheck

** Calculation of lottery index: mlottery[1,1]

matrix mrcsign = mat1sqr

forvalues hpr=1(1)$hpnum {

forvalues hpc=1(1)$hpnum {

matrix mrcsign[`hpr',`hpc']=sign(mrcpqd[`hpr',`hpc'])

}

}

matrix mat1=hadamard(mrcsign, mrcpqd)

matrix mat2 = matwgt'*mat1*matwgt

matrix mat1= matwgt'*matwgt

scalar normfactor =mat1[1,1]

matrix mlottery = mat2/(1-normfactor)

matrix list mlottery

**Appendix 2. Selected distribution regression model results**

Tables A2.1 through A2.6 provide fixed effects estimates of the linear probability distribution regression (LPDRM) and generalised linear distribution regression (GLDRM) models for the GPPS, CQC and QOF practice quality indicators.

Table A2.1: LPDRM for GPPS 5-category patient experience responses (full GPPS sample)

Table A2.2: GLDRM for GPPS 5-category patient experience responses (full GPPS sample)

Table A2.3: LPDRM for CQC 4-category inspection ratings (matched sample)

Table A2.4: GLDRM for CQC 4-category inspection ratings (matched sample)

Table A2.5: LPDRM for 5-category grouping of total QOF score (matched sample)

Table A2.6: GLDRM for 5-category grouping of total QOF score (matched sample)

Table A2.1 Fixed effects estimates of the LPDRM for GPPS 5-category indicator (full GPPS sample)

|  | Reported experience no better than: | | | | | | | | | | |  | |
| --- | --- | --- | --- | --- | --- | --- | --- | --- | --- | --- | --- | --- | --- |
| *Dependent variable* | very poor  *P*(*q*≤*1*) | | | fairly poor  *P*(*q*≤*2*) | | | neither good nor poor  *P(q≤3)* | | | fairly good  *I(q≤4)* | | | |
| *FEMALE* | -0.0112 |  | -0.0043 | |  | -0.0455 | |  | -0.0063 | |  | |  |
|  | *0.0065* |  | *0.0145* | |  | *0.0241* | |  | *0.0314* | |  | |  |
| *AGE 16-24* | -0.0404 | ^**^ | -0.0656 | | ^**^ | -0.0403 | |  | 0.0216 | |  | |  |
|  | *0.0085* |  | *0.0187* | |  | *0.0329* | |  | *0.0500* | |  | |  |
| *AGE 25-34* | -0.0132 |  | -0.0222 | |  | 0.0108 | |  | 0.1102 | | ^*^ | |  |
|  | *0.0099* |  | *0.0192* | |  | *0.0333* | |  | *0.0477* | |  | |  |
| *AGE 35-44* | -0.0083 |  | -0.0016 | |  | 0.0303 | |  | 0.1511 | | ^**^ | |  |
|  | *0.0108* |  | *0.0212* | |  | *0.0350* | |  | *0.0536* | |  | |  |
| *AGE 55-64* | -0.0382 | ^**^ | -0.0820 | | ^**^ | -0.1216 | | ^**^ | -0.0841 | |  | |  |
|  | *0.0122* |  | *0.0256* | |  | *0.0433* | |  | *0.0630* | |  | |  |
| *AGE 65-74* | -0.0513 | ^**^ | -0.1363 | | ^**^ | -0.2440 | | ^**^ | -0.3252 | | ^**^ | |  |
|  | *0.0163* |  | *0.0331* | |  | *0.0564* | |  | *0.0792* | |  | |  |
| *AGE 75-84* | -0.0587 | ^**^ | -0.1532 | | ^**^ | -0.1360 | |  | -0.1507 | |  | |  |
|  | *0.0202* |  | *0.0509* | |  | *0.0802* | |  | *0.1056* | |  | |  |
| *AGE 85+* | -0.0700 | ^*^ | -0.1578 | | ^*^ | -0.2954 | | ^**^ | -0.3503 | | ^*^ | |  |
|  | *0.0289* |  | *0.0619* | |  | *0.1010* | |  | *0.1492* | |  | |  |
| *BLACK* | 0.0127 |  | -0.0146 | |  | 0.0643 | |  | 0.1547 | |  | |  |
|  | *0.0208* |  | *0.0415* | |  | *0.0745* | |  | *0.1075* | |  | |  |
| *ASIAN* | 0.0410 | ^**^ | 0.0700 | | ^**^ | 0.1302 | | ^**^ | 0.1751 | | ^**^ | |  |
|  | *0.0033* |  | *0.0077* | |  | *0.0127* | |  | *0.0178* | |  | |  |
| *MIXED* | 0.0139 |  | 0.0121 | |  | 0.0198 | |  | 0.0418 | |  | |  |
|  | *0.0081* |  | *0.0151* | |  | *0.0310* | |  | *0.0437* | |  | |  |
| *OTHER* | 0.0114 |  | 0.0104 | |  | 0.0216 | |  | 0.0399 | |  | |  |
|  | *0.0156* |  | *0.0282* | |  | *0.0487* | |  | *0.0691* | |  | |  |
| *LONG TERM CONDITION* | 0.0380 | ^**^ | 0.1040 | | ^**^ | 0.1653 | | ^**^ | 0.2608 | | ^**^ | |  |
|  | *0.0066* |  | *0.0132* | |  | *0.0243* | |  | *0.0350* | |  | |  |
| *IMD SCORE* | 0.0002 | ^**^ | 0.0005 | | ^**^ | 0.0010 | | ^**^ | 0.0013 | | ^**^ | |  |
|  | *0.0000* |  | *0.0001* | |  | *0.0001* | |  | *0.0002* | |  | |  |
| *constant* | 0.0207 | ^*^ | 0.0384 | | ^*^ | 0.1124 | | ^**^ | 0.3390 | | ^**^ | |  |
|  | *0.0084* |  | *0.0171* | |  | *0.0300* | |  | *0.0451* | |  | |  |
| *Practices* | 6926 |  | 6926 | |  | 6926 | |  | 6926 | |  | |  |
| *CCG clusters* | 195 |  | 195 | |  | 195 | |  | 195 | |  | |  |
| *R^2^* | 0.205 |  | 0.213 | |  | 0.254 | |  | 0.258 | |  | |  |
| *RMSE* | 0.024 |  | 0.050 | |  | 0.086 | |  | 0.125 | |  | |  |

*Notes: The dependent variable P(q≤c) takes a value equal to the proportion of patients in a practice reporting their experience as no better than category c (c=1,2,3,4). Positive (negative) coefficients imply higher (lower) proportions than for the reference group of White men aged 45-54 not reporting a long-term health condition and registered at a practice with an IMD score of zero in NHS Darlington CCG: for example, a 1pp increase in the proportion of Asian patients is predicted to lead to ceteris paribus increases of 0.0410 pp, 0.0700 pp, 0.1302pp and 0.1751pp in the proportions reporting experience no better than very poor, fairly poor, neither poor nor good, and fairly good respectively. CCG fixed effects not reported. Robust CCG-clustered standard errors are in italics. *p<0.05, **p<0.01. Source: Own calculations from GPPS data.*

Table A2.2 Fixed effects estimates of GLDRM for GPPS 5-category indicator (full GPPS sample)

|  | Reported experience no better than: | | | | | | | | | | |  | |
| --- | --- | --- | --- | --- | --- | --- | --- | --- | --- | --- | --- | --- | --- |
|  | very poor  *P*(*q*≤*1*) | | | fairly poor  *P*(*q*≤*2*) | | | neither good nor poor  *P(q≤3)* | | | fairly good  *I(q≤4)* | | | |
| *FEMALE* | -0.1937 |  | -0.0533 | |  | -0.1757 | |  | -0.0125 | |  | |  |
|  | *0.1322* |  | *0.1158* | |  | *0.0995* | |  | *0.0832* | |  | |  |
| *AGE 16-24* | -0.6491 | ^**^ | -0.4187 | | ^*^ | -0.0431 | |  | 0.0970 | |  | |  |
|  | *0.2003* |  | *0.1670* | |  | *0.1428* | |  | *0.1314* | |  | |  |
| *AGE 25-34* | -0.2066 |  | -0.1379 | |  | 0.0474 | |  | 0.2751 | | ^*^ | |  |
|  | *0.1913* |  | *0.1565* | |  | *0.1390* | |  | *0.1261* | |  | |  |
| *AGE 35-44* | -0.0855 |  | 0.0268 | |  | 0.1675 | |  | 0.4044 | | ^**^ | |  |
|  | *0.2089* |  | *0.1688* | |  | *0.1436* | |  | *0.1411* | |  | |  |
| *AGE 55-64* | -0.6994 | ^**^ | -0.6185 | | ^**^ | -0.4747 | | ^**^ | -0.2369 | |  | |  |
|  | *0.2454* |  | *0.2039* | |  | *0.1775* | |  | *0.1675* | |  | |  |
| *AGE 65-74* | -1.2100 | ^**^ | -1.2191 | | ^**^ | -1.0665 | | ^**^ | -0.8944 | | ^**^ | |  |
|  | *0.3346* |  | *0.2679* | |  | *0.2296* | |  | *0.2053* | |  | |  |
| *AGE 75-84* | -1.1772 | ^**^ | -1.1883 | | ^**^ | -0.5557 | |  | -0.3920 | |  | |  |
|  | *0.4391* |  | *0.4326* | |  | *0.3390* | |  | *0.2761* | |  | |  |
| *AGE 85+* | -1.4932 | ^*^ | -1.2880 | | ^*^ | -1.2429 | | ^**^ | -0.9641 | | ^*^ | |  |
|  | *0.6588* |  | *0.5537* | |  | *0.4356* | |  | *0.3940* | |  | |  |
| *BLACK* | 0.3651 |  | -0.0126 | |  | 0.1528 | |  | 0.2858 | |  | |  |
|  | *0.3966* |  | *0.3127* | |  | *0.2731* | |  | *0.2673* | |  | |  |
| *ASIAN* | 0.5453 | ^**^ | 0.4160 | | ^**^ | 0.4131 | | ^**^ | 0.4672 | | ^**^ | |  |
|  | *0.0543* |  | *0.0534* | |  | *0.0478* | |  | *0.0490* | |  | |  |
| *MIXED* | 0.1555 |  | -0.0066 | |  | 0.0094 | |  | 0.0766 | |  | |  |
|  | *0.1390* |  | *0.1080* | |  | *0.1164* | |  | *0.1156* | |  | |  |
| *OTHER* | 0.2491 |  | 0.1555 | |  | 0.1874 | |  | 0.2359 | |  | |  |
|  | *0.2538* |  | *0.1991* | |  | *0.1813* | |  | *0.1837* | |  | |  |
| *LONG TERM CONDITION* | 0.8567 | ^**^ | 0.9057 | | ^**^ | 0.7359 | | ^**^ | 0.7317 | | ^**^ | |  |
|  | *0.1251* |  | *0.1048* | |  | *0.0987* | |  | *0.0909* | |  | |  |
| *IMD SCORE* | 0.0039 | ^**^ | 0.0036 | | ^**^ | 0.0040 | | ^**^ | 0.0033 | | ^**^ | |  |
|  | *0.0006* |  | *0.0006* | |  | *0.0005* | |  | *0.0004* | |  | |  |
| *constant* | -2.1558 | ^**^ | -1.7971 | | ^**^ | -1.2587 | | ^**^ | -0.4548 | | ^**^ | |  |
|  | *0.1621* |  | *0.1345* | |  | *0.1239* | |  | *0.1194* | |  | |  |
| *Practices* | 6926 |  | 6926 | |  | 6926 | |  | 6926 | |  | |  |
| *CCG clusters* | 195 |  | 195 | |  | 195 | |  | 195 | |  | |  |

*Notes: The dependent variable P(q≤c) takes a value equal to the proportion of patients in a practice reporting their experience as no better than category c (c=1,2,3,4). CCG fixed effects not reported. Semirobust CCG-clustered standard errors are in italics. *p<0.05, **p<0.01. Source: Own calculations from GPPS data.*

Table A2.3 Fixed effects estimates of the LPDRM: CQC 4-category (matched sample)

|  | Inspection rating no better than: | | | | | | | | | |
| --- | --- | --- | --- | --- | --- | --- | --- | --- | --- | --- |
|  | inadequate  *P*(*q*≤*1*) | | | requires improvement  *P*(*q*≤*2*) | | | good  *P(q≤3)* | | |  |
| *FEMALE* | -0.0013 |  | -0.0442 | |  | -0.1536 | | ^*^ |  |  |
|  | *0.0263* |  | *0.0431* | |  | *0.0782* | |  |  |  |
| *AGE 16-24* | 0.0303 |  | 0.0457 | |  | -0.1243 | |  |  |  |
|  | *0.0405* |  | *0.0737* | |  | *0.1773* | |  |  |  |
| *AGE 25-34* | -0.0085 |  | 0.0492 | |  | -0.1493 | |  |  |  |
|  | *0.0334* |  | *0.0751* | |  | *0.1186* | |  |  |  |
| *AGE 35-44* | -0.0057 |  | 0.0011 | |  | -0.0882 | |  |  |  |
|  | *0.0354* |  | *0.0751* | |  | *0.1243* | |  |  |  |
| *AGE 55-64* | -0.0457 |  | 0.0561 | |  | 0.1352 | |  |  |  |
|  | *0.0361* |  | *0.0935* | |  | *0.1377* | |  |  |  |
| *AGE 65-74* | -0.0109 |  | -0.1399 | |  | 0.0599 | |  |  |  |
|  | *0.0447* |  | *0.1277* | |  | *0.1604* | |  |  |  |
| *AGE 75-84* | 0.0052 |  | 0.0546 | |  | -0.1662 | |  |  |  |
|  | *0.0508* |  | *0.1265* | |  | *0.1957* | |  |  |  |
| *AGE 85+* | 0.1257 |  | 0.3135 | |  | -0.4235 | |  |  |  |
|  | *0.1158* |  | *0.2082* | |  | *0.3654* | |  |  |  |
| *BLACK* | 0.0078 |  | -0.1828 | |  | 0.2313 | |  |  |  |
|  | *0.0697* |  | *0.1251* | |  | *0.2274* | |  |  |  |
| *ASIAN* | 0.0268 |  | 0.0716 | | ^*^ | 0.0719 | | ^*^ |  |  |
|  | *0.0194* |  | *0.0332* | |  | *0.0332* | |  |  |  |
| *MIXED* | 0.0127 |  | 0.1101 | |  | -0.1513 | | ^*^ |  |  |
|  | *0.0223* |  | *0.0647* | |  | *0.0751* | |  |  |  |
| *OTHER* | 0.0368 |  | 0.0046 | |  | 0.1602 | |  |  |  |
|  | *0.0449* |  | *0.0924* | |  | *0.0962* | |  |  |  |
| *LONG TERM CONDITION* | 0.0127 |  | -0.0028 | |  | 0.0712 | |  |  |  |
|  | *0.0192* |  | *0.0382* | |  | *0.0582* | |  |  |  |
| *IMD SCORE* | 0.0000 |  | 0.0003 | |  | 0.0007 | |  |  |  |
|  | *0.0001* |  | *0.0002* | |  | *0.0004* | |  |  |  |
| *constant* | -0.0045 |  | -0.0017 | |  | 0.9872 | | ^**^ |  |  |
|  | *0.0329* |  | *0.0646* | |  | *0.1004* | |  |  |  |
| *Practices* | 6427 |  | 6427 | |  | 6427 | |  |  |  |
| *CCG clusters* | 195 |  | 195 | |  | 195 | |  |  |  |
| *R^2^* | 0.044 |  | 0.060 | |  | 0.117 | |  |  |  |
| *RMSE* | 0.084 |  | 0.183 | |  | 0.227 | |  |  |  |

*Notes: The dependent variable P(q≤c) takes a value of one if practice quality is no better than category c (c=1,2,3) and zero otherwise. Positive (negative) coefficients imply higher (lower) chances than for the reference group of White men aged 45-54 not reporting a long-term health condition and registered at a practice with an IMD score of zero in NHS Darlington CCG: for example, a 1pp increase in the proportion of Asian patients is predicted to lead to ceteris paribus increases of 0.0268pp, 0.0716pp, and 0.0719pp in the chances of an inspection rating no better than inadequate, requires improvement, and good respectively. CCG fixed effects not reported. Robust CCG-clustered standard errors are in italics. *p<0.05, **p<0.01. Source: Own calculations from CQC data.*

Table A2.4 Fixed effects estimates of the GLDRM: CQC 4-category (matched sample)

|  | Inspection rating no better than: | | | | | | | | | |
| --- | --- | --- | --- | --- | --- | --- | --- | --- | --- | --- |
|  | inadequate  *P*(*q*≤*1*) | | | requires improvement  *P*(*q*≤*2*) | | | good  *P(q≤3)* | | |  |
| *FEMALE* | -0.4299 |  | -0.5800 | |  | -1.4296 | | ^*^ |  |  |
|  | *1.1746* |  | *0.5955* | |  | *0.6902* | |  |  |  |
| *AGE 16-24* | 1.0801 |  | 0.7261 | |  | -0.8120 | |  |  |  |
|  | *1.7195* |  | *0.9770* | |  | *1.2984* | |  |  |  |
| *AGE 25-34* | -0.7409 |  | 0.8462 | |  | -1.3819 | |  |  |  |
|  | *1.9944* |  | *1.0452* | |  | *1.1516* | |  |  |  |
| *AGE 35-44* | -0.3825 |  | 0.1227 | |  | -1.4327 | |  |  |  |
|  | *2.1615* |  | *1.0148* | |  | *1.3188* | |  |  |  |
| *AGE 55-64* | -3.8790 |  | 0.8596 | |  | 1.5434 | |  |  |  |
|  | *2.4327* |  | *1.3323* | |  | *1.5350* | |  |  |  |
| *AGE 65-74* | -0.6664 |  | -1.8767 | |  | 0.6885 | |  |  |  |
|  | *2.9592* |  | *1.8504* | |  | *1.7524* | |  |  |  |
| *AGE 75-84* | 0.5996 |  | 0.8649 | |  | -2.0839 | |  |  |  |
|  | *3.4266* |  | *1.9285* | |  | *1.9541* | |  |  |  |
| *AGE 85+* | 7.4179 |  | 4.9453 | |  | -3.7015 | |  |  |  |
|  | *7.4871* |  | *3.2013* | |  | *3.1760* | |  |  |  |
| *BLACK* | 1.1508 |  | -2.0299 | |  | 1.5457 | |  |  |  |
|  | *2.9108* |  | *1.5228* | |  | *2.5232* | |  |  |  |
| *ASIAN* | 1.0497 |  | 0.7010 | | ^*^ | 1.1485 | | ^*^ |  |  |
|  | *0.5908* |  | *0.3085* | |  | *0.4961* | |  |  |  |
| *MIXED* | 0.6627 |  | 1.0557 | |  | -2.2886 | | ^*^ |  |  |
|  | *0.7729* |  | *0.5732* | |  | *1.1014* | |  |  |  |
| *OTHER* | 1.9242 |  | 0.3070 | |  | 2.8149 | |  |  |  |
|  | *1.3086* |  | *0.8943* | |  | *1.5997* | |  |  |  |
| *LONG TERM CONDITION* | 0.8459 |  | 0.1714 | |  | 0.9006 | |  |  |  |
|  | *1.0767* |  | *0.5278* | |  | *0.6041* | |  |  |  |
| *IMD SCORE* | 0.0070 |  | 0.0054 | |  | 0.0052 | |  |  |  |
|  | *0.0064* |  | *0.0032* | |  | *0.0036* | |  |  |  |
| *constant* | -5.0671 | ^**^ | -5.1657 | | ^**^ | 1.9569 | | ^*^ |  |  |
|  | *1.8250* |  | *0.9395* | |  | *0.9508* | |  |  |  |
| *Practices* | 6427 |  | 6427 | |  | 6427 | |  |  |  |
| *CCG clusters* | 195 |  | 195 | |  | 195 | |  |  |  |

*Notes: The dependent variable P(q≤c) takes a value of one if practice quality is no better than category c (c=1,2,3) and zero otherwise. CCG fixed effects not reported. Semirobust CCG-clustered standard errors are in italics. *p<0.05, **p<0.01. Source: Own calculations from CQC data.*

Table A2.5 Fixed effects estimates of the LPDRM: 5-category grouping of QOF score (matched sample)

|  | Reported experience no better than: | | | | | | | | | | |  | |
| --- | --- | --- | --- | --- | --- | --- | --- | --- | --- | --- | --- | --- | --- |
|  | Category1  *P*(*q*≤*1*) | | | Category2  *P*(*q*≤*2*) | | | Category3  *P(q≤3)* | | | Category4  *I(q≤4)* | | | |
| *FEMALE* | 0.0687 |  | -0.0777 | |  | -0.0938 | |  | -0.1327 | |  | |  |
|  | *0.0474* |  | *0.0728* | |  | *0.1101* | |  | *0.1309* | |  | |  |
| *AGE 16-24* | 0.3731 | ^*^ | 0.4695 | | ^**^ | 0.5776 | | ^**^ | 0.3598 | | ^*^ | |  |
|  | *0.1498* |  | *0.1590* | |  | *0.1676* | |  | *0.1787* | |  | |  |
| *AGE 25-34* | 0.0624 |  | 0.0553 | |  | 0.1054 | |  | 0.2423 | |  | |  |
|  | *0.0477* |  | *0.0953* | |  | *0.1525* | |  | *0.1765* | |  | |  |
| *AGE 35-44* | 0.0697 |  | 0.1792 | |  | 0.0077 | |  | 0.0906 | |  | |  |
|  | *0.0469* |  | *0.1033* | |  | *0.1486* | |  | *0.2325* | |  | |  |
| *AGE 55-64* | -0.0289 |  | -0.0066 | |  | -0.0838 | |  | -0.0471 | |  | |  |
|  | *0.0608* |  | *0.1342* | |  | *0.1547* | |  | *0.2631* | |  | |  |
| *AGE 65-74* | 0.0466 |  | 0.0673 | |  | 0.0205 | |  | 0.4584 | |  | |  |
|  | *0.1077* |  | *0.1530* | |  | *0.2421* | |  | *0.3103* | |  | |  |
| *AGE 75-84* | 0.0132 |  | -0.0623 | |  | -0.3636 | |  | -0.8810 | | ^*^ | |  |
|  | *0.0697* |  | *0.1999* | |  | *0.3090* | |  | *0.3546* | |  | |  |
| *AGE 85+* | -0.0680 |  | -0.0700 | |  | 0.2433 | |  | -0.2646 | |  | |  |
|  | *0.1028* |  | *0.2395* | |  | *0.4049* | |  | *0.5140* | |  | |  |
| *BLACK* | 0.4833 |  | 0.4418 | |  | 0.7000 | | ^*^ | 1.1272 | | ^**^ | |  |
|  | *0.3162* |  | *0.3383* | |  | *0.3112* | |  | *0.3992* | |  | |  |
| *ASIAN* | -0.0267 |  | -0.0771 | |  | -0.0644 | |  | 0.1074 | |  | |  |
|  | *0.0214* |  | *0.0395* | |  | *0.0591* | |  | *0.0723* | |  | |  |
| *MIXED* | 0.0624 |  | 0.1380 | |  | 0.1223 | |  | 0.5005 | | ^**^ | |  |
|  | *0.0469* |  | *0.0869* | |  | *0.1534* | |  | *0.1674* | |  | |  |
| *OTHER* | -0.0763 |  | 0.0155 | |  | 0.0870 | |  | 0.0500 | |  | |  |
|  | *0.0610* |  | *0.1509* | |  | *0.1992* | |  | *0.2438* | |  | |  |
| *LONG TERM CONDITION* | -0.0027 |  | 0.0407 | |  | -0.0179 | |  | 0.0654 | |  | |  |
|  | *0.0389* |  | *0.0608* | |  | *0.0874* | |  | *0.1156* | |  | |  |
| *IMD SCORE* | -0.0002 |  | 0.0004 | |  | 0.0008 | |  | 0.0016 | | ^**^ | |  |
|  | *0.0001* |  | *0.0004* | |  | *0.0005* | |  | *0.0006* | |  | |  |
| *constant* | -0.0849 |  | -0.0728 | |  | -0.0033 | |  | 0.1547 | |  | |  |
|  | *0.0552* |  | *0.0886* | |  | *0.1185* | |  | *0.1733* | |  | |  |
| *Practices* | 6427 |  | 6427 | |  | 6427 | |  | 6427 | |  | |  |
| *CCG clusters* | 195 |  | 195 | |  | 195 | |  | 195 | |  | |  |
| *R^2^* | 0.318 |  | 0.256 | |  | 0.181 | |  | 0.160 | |  | |  |
| *RMSE* | 0.118 |  | 0.215 | |  | 0.346 | |  | 0.464 | |  | |  |

*Notes: The dependent variable P(q≤c) takes a value of one if practice quality is no better than category c (c=1,2,3,4) and zero otherwise. Grouping of data into categories based on English proportions for GPPS 5-category indicator. Positive (negative) coefficients imply higher (lower) chances than for the reference group of White men aged 45-54 not reporting a long-term health condition and registered at a practice with an IMD score of zero in NHS Darlington CCG: for example, a 1 pp increase in the proportion of Asian patients is predicted to lead to ceteris paribus reductions of 0.0267pp, 0.0771pp, and 0.0644pp in the chances of a QOF score no better than category 1, 2 and 3 respectively, and an increase of 0.1074pp in the probability of a score no better than category 4. CCG fixed effects not reported. Robust CCG-clustered standard errors are in italics. *p<0.05, **p<0.01. Source: Own calculations from QOF data.*

Table A2.6 Fixed effects estimates of the GLDRM: 5-category grouping of QOF score (matched sample)

|  | Reported experience no better than: | | | | | | | | | | |  | |
| --- | --- | --- | --- | --- | --- | --- | --- | --- | --- | --- | --- | --- | --- |
|  | Category1  *P*(*q*≤*1*) | | | Category2  *P*(*q*≤*2*) | | | Category3  *P(q≤3)* | | | Category4  *I(q≤4)* | | | |
| *FEMALE* | -0.0464 |  | 0.6135 | |  | -0.4402 | |  | -0.3433 | |  | |  |
|  | *0.8597* |  | *-1.9900* | |  | *0.4712* | |  | *0.3746* | |  | |  |
| *AGE 16-24* | 5.2101 | ^**^ | 3.3669 | | ^**^ | 1.9573 | | ^**^ | 1.1021 | | ^*^ | |  |
|  | *1.3960* |  | *1.0132* | |  | *0.6234* | |  | *0.5457* | |  | |  |
| *AGE 25-34* | 0.4675 |  | 0.2397 | |  | 0.2547 | |  | 0.7042 | |  | |  |
|  | *1.6832* |  | *0.9896* | |  | *0.6447* | |  | *0.5159* | |  | |  |
| *AGE 35-44* | 2.7908 |  | 1.7800 | |  | 0.0145 | |  | 0.2287 | |  | |  |
|  | *1.8421* |  | *1.0821* | |  | *0.6830* | |  | *0.6832* | |  | |  |
| *AGE 55-64* | -1.8155 |  | 0.1103 | |  | -0.4146 | |  | -0.0968 | |  | |  |
|  | *2.7382* |  | *1.4695* | |  | *0.7257* | |  | *0.7571* | |  | |  |
| *AGE 65-74* | 0.7016 |  | 0.4342 | |  | -0.1504 | |  | 1.3977 | |  | |  |
|  | *4.3051* |  | *1.8103* | |  | *1.1588* | |  | *0.8895* | |  | |  |
| *AGE 75-84* | -1.2960 |  | -1.5713 | |  | -2.2161 | |  | -2.5389 | | ^*^ | |  |
|  | *2.6815* |  | *2.3386* | |  | *1.5180* | |  | *1.0288* | |  | |  |
| *AGE 85+* | -3.9137 |  | -1.1923 | |  | 1.1826 | |  | -0.8590 | |  | |  |
|  | *3.5191* |  | *3.0106* | |  | *2.1009* | |  | *1.4790* | |  | |  |
| *BLACK* | 4.9822 |  | 2.4698 | |  | 2.4615 | | ^*^ | 3.6204 | | ^**^ | |  |
|  | *2.7893* |  | *1.8588* | |  | *1.1173* | |  | *1.3361* | |  | |  |
| *ASIAN* | -0.3407 |  | -0.5493 | |  | -0.2039 | |  | 0.2849 | |  | |  |
|  | *0.6877* |  | *0.3810* | |  | *0.2325* | |  | *0.2099* | |  | |  |
| *MIXED* | 2.8189 | ^**^ | 0.9322 | |  | 0.3743 | |  | 1.5978 | | ^**^ | |  |
|  | *0.9968* |  | *0.6079* | |  | *0.5494* | |  | *0.5202* | |  | |  |
| *OTHER* | -0.5216 |  | 0.0462 | |  | 0.2850 | |  | 0.1214 | |  | |  |
|  | *2.1754* |  | *1.0265* | |  | *0.7029* | |  | *0.7276* | |  | |  |
| *LONG TERM CONDITION* | 0.4995 |  | 0.5838 | |  | 0.0038 | |  | 0.1444 | |  | |  |
|  | *1.0429* |  | *0.6157* | |  | *0.3842* | |  | *0.3297* | |  | |  |
| *IMD SCORE* | -0.0117 |  | 0.0050 | |  | 0.0042 | | ^*^ | 0.0045 | | ^**^ | |  |
|  | *0.0071* |  | *0.0037* | |  | *0.0020* | |  | *0.0016* | |  | |  |
| *constant* | -5.6071 | ^**^ | -5.2793 | | ^**^ | -4.5496 | | ^**^ | -0.9587 | |  | |  |
|  | *1.5549* |  | *0.9573* | |  | *0.5831* | |  | *0.5015* | |  | |  |
| *Practices* | 6427 |  | 6427 | |  | 6427 | |  | 6427 | |  | |  |
| *CCG clusters* | 195 |  | 195 | |  | 195 | |  | 195 | |  | |  |

*Notes: The dependent variable P(q≤c) takes a value of one if practice quality is no better than category c (c=1,2,3,4) and zero otherwise. Grouping of data into categories based on English proportions for GPPS 5-category indicator. CCG fixed effects not reported. Semirobust CCG-clustered standard errors are in italics. *p<0.05, **p<0.01. Source: Own calculations from QOF data.*
